# Visualization of the process of a nanocarrier-mediated gene delivery: stabilization, endocytosis and endosomal escape of genes for intracellular spreading

**DOI:** 10.1186/s12951-022-01336-6

**Published:** 2022-03-09

**Authors:** Zhongzheng Ma, Yang Zheng, Zijian Chao, Hongtao Chen, Yunhui Zhang, Meizhen Yin, Jie Shen, Shuo Yan

**Affiliations:** 1 Department of Plant Biosecurity and MOA Key Laboratory of Pest Monitoring and Green Management, College of Plant Protection, China Agricultural University, Beijing, 100193 People’s Republic of China; 2 Institute of Plant Protection, Beijing Academy of Agriculture and Forestry Sciences, Beijing, 100097 People’s Republic of China; 3 College of Horticulture and Plant Protection, Yangzhou University, Yangzhou, 225002 Jiangsu People’s Republic of China; 4 State Key Laboratory of Chemical Resource Engineering, Beijing Lab of Biomedical Materials, Beijing University of Chemical Technology, Beijing, 100029 People’s Republic of China

**Keywords:** Cellular uptake, Clathrin, Endocytosis, dsRNA, Nanocarrier, RNA interference

## Abstract

Nanoparticles have been widely applied as gene carrier for improving RNA interference (RNAi) efficiency in medical and agricultural fields. However, the mechanism and delivery process of nanoparticle-mediated RNAi is not directly visualized and elucidated. Here we synthesized a star polymer (SPc) consisted of a hydrophilic shell with positively-charged tertiary amine in the side chain, which was taken as an example to investigate the mechanism in gene delivery. The SPc could assemble with dsRNA spontaneously through electrostatic force, hydrogen bond and van der Waals force. Interestingly, the SPc could protect dsRNA from degradation by RNase A and insect hemolymph, thus remarkably increasing the stability of dsRNA. Meanwhile, the SPc could efficiently promote the cellular uptake and endosomal escape for intracellular spreading of dsRNA. Transcriptome analysis revealed that the SPc could up-regulate some key genes such as *Chc*, *AP2S1* and *Arf1* for activating clathrin-mediated endocytosis. Furthermore, the suppression of endocytosis hindered the cellular uptake of SPc-delivered dsRNA in vitro, and the subsequent RNAi effect was also disappeared in vivo**.** To our knowledge, our study is the first direct visualization of the detailed cellular delivery process and mechanism of nanocarrier-mediated gene delivery. Above mechanism supports the application of nanocarrier-based RNAi in gene therapy and pest management.

**Graphical Abstract:**

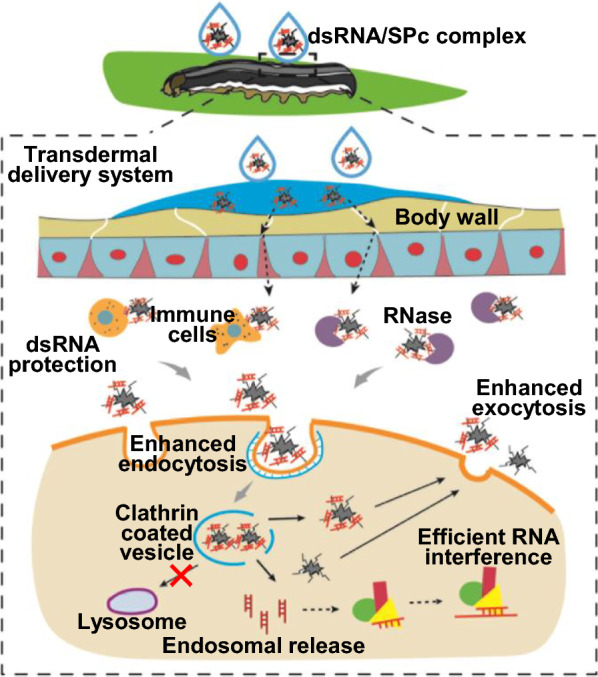

**Supplementary Information:**

The online version contains supplementary material available at 10.1186/s12951-022-01336-6.

## Introduction

RNA interference (RNAi) is widely used to interfere gene function and shows great potential for gene therapy and pest management in medical and agricultural fields [1–5]. However the major limitation of small interfering RNA (siRNA) or double-stranded RNA (dsRNA) is the inability to passively diffuse through cellular membrane due to the electrostatic repulsion from the anionic cell membrane surface [6, 7]. Meanwhile, siRNA/dsRNA is highly susceptible to nuclease-induced degradation [6–8]. In recent years, RNAi has been recognized as a novel and safe strategy in pest green management [9–12]. However, RNAi efficiency varies greatly among different insect species, and the major limitations for efficient RNAi include dsRNA instability, low efficiency of dsRNA cellular internalization, deficient core RNAi machinery and impaired systemic spreading of dsRNA, which constrains the application of RNAi-based pest management [13–18].

Nano-delivery systems have been bloomed over the past 30 years, and several types of synthetic gene vectors such as cationic lipids, chitosan and quantum dots have been designed and constructed to overcome the delivery obstacle for efficient RNAi [19–23]. Nanocarrier-mediated RNAi has been tested in several insect species, and high RNAi efficiency has been observed in African malaria mosquito *Anopheles gambiae* [24], Yellow fever mosquito *Aedes aegypti* [25], German cockroach *Blattella germanica* [26], fall armyworm *Spodoptera frugiperda* [27] and beet armyworm *S.* *exigua* [28]. Previous studies have implied that the application of nano-delivery system might solve the systemic and intracellular barriers such as rapid degradation and poor cellular uptake [29–33]. Based on current knowledge, receptor-mediated endocytosis may be the major route for nanocarriers to enter into the cells [34–36]. Nanocarriers are usually coated by membrane-bound vesicles called endosomes following cellular uptake, and the late endosomes ultimately fuse with degradative lysosomes [37, 38]. Therefore, endosomal escape has become another key step for nano-delivery systems [39]. However, the detailed delivery process and mechanism of nanoparticle-mediated RNAi have not been directly visualized and elucidated, which leads heated discussion to constrain its further application in agricultural field.

Our group has constructed a nanocarrier-based platform for pest management [40–43]. A facile-synthesized star polycation (SPc) is designed to construct a transdermal dsRNA delivery system for controlling aphids, which has been selected for Research Highlights from China collection by Springer Nature [44, 45]. Subsequently, the SPc-delivered dsRNA is applied to disrupt the wing development of fruit flies and green peach aphids and inhibit the feeding behavior of oriental fruit moths [46–48]. So far, the SPc has been widely shared with researchers in China, and the transdermal dsRNA delivery system has been successfully applied in more than 30 insect species. The current study focused on the mechanism and delivery process of SPc-mediated RNAi. We determined the interaction of SPc with dsRNA, tested the stability of SPc-complexed dsRNA, investigated the cellular uptake and intracellular fate of dsRNA/SPc complex, and finally confirmed the major gene pathway for the cellular uptake of dsRNA/SPc complex. Our study directly visualized and elucidated the detailed cellular process and mechanism of polymer-mediated RNAi, which supports the development and practice of RNAi-based gene therapy and pest management.

## Results and discussion

### Loading capacity of SPc and its interaction with dsRNA

**Fig. 1:**
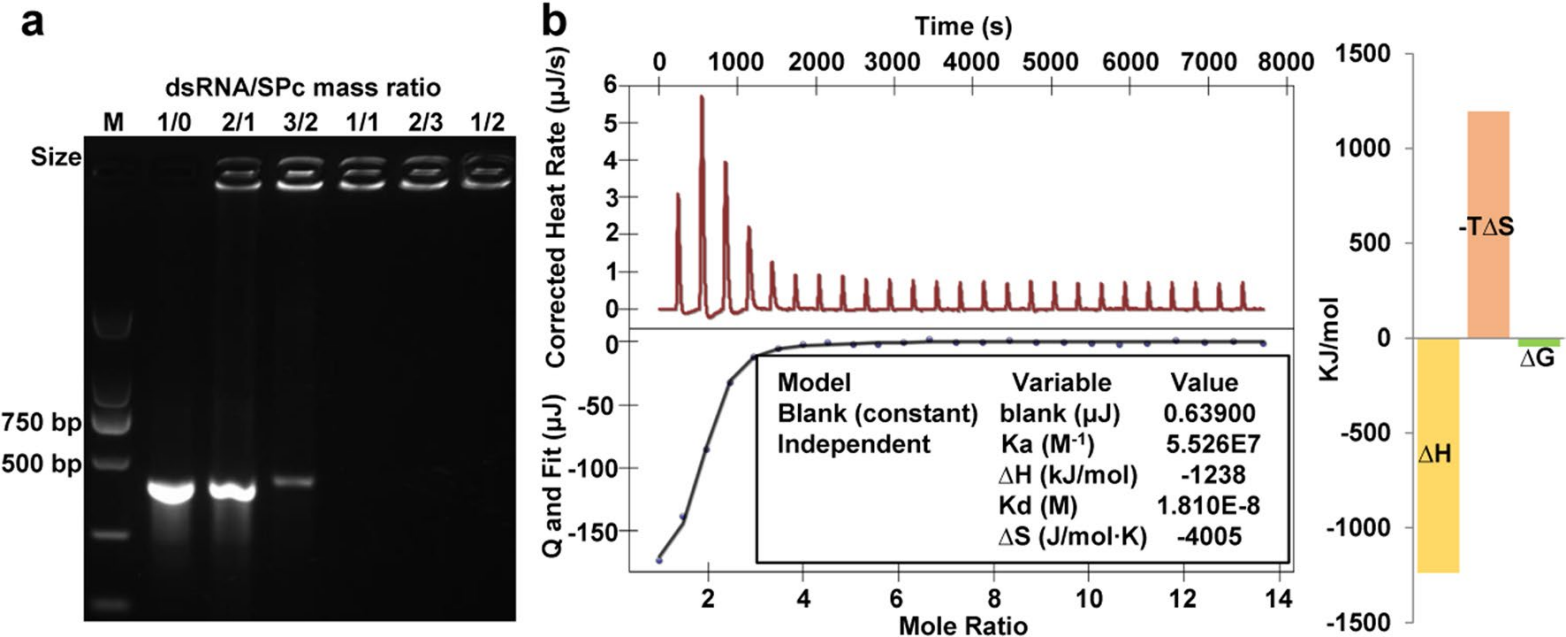
Self-assembly mechanism of dsRNA/SPc complex. **a** Gel electrophoresis assay of ds*eGFP* retardation by SPc. One μg ds*eGFP* was mixed with SPc at various mass ratios, and the mixture (10 μL) was incubated and then analyzed. M: DNA marker. **b** ITC titration of ds*eGFP* (0.333 μM) into SPc solution (5.1 μM). The heats of interaction during each injection were calculated by the integration of each titration peak. The test temperature was 25 °C

The SPc was synthesized using commercially available pentaerythritol to construct the star initiator Pt-Br, which was further polymerized with DMAEMA. The solvent THF was removed, and dialysis was then conducted to purify the crude product. The SPc was finally obtained as white powder after freeze-drying (Additional file 1: Fig. S1). The SPc is consisted of a hydrophobic core and a hydrophilic shell with positively-charged tertiary amine in the side chain, and the particle size of SPc is 100.5 nm with zeta potential of 20.9 mV [44]. The SPc was firstly used as a gene vector for efficient dsRNA delivery, achieving good gene silencing effects on insects [45–48]. However, the interactions between SPc and dsRNA were not very clear. To investigate the loading capacity of SPc toward dsRNA, ds*eGFP* was mixed with SPc at various mass ratios and analyzed using gel retardation test. As shown in Fig. 1a, the band’s intensity of the migrated ds*eGFP* gradually decreased with the increasing mass ratios, indicating that the SPc had excellent performance in combining with dsRNA. Considering the electrostatic interaction of tertiary amines with dsRNA [49, 50], we deduced that the tertiary amine in SPc might combine with negatively-charged nucleic acids through electrostatic interaction, which resulted in the electronegativity loss of dsRNA. Our unpublished data demonstrated that the particle size of ds*eGFP*/SPc complex (221.39 nm) was bigger than that of SPc, and its zeta potential was decreased to 0.78 mV, suggesting the potential electrostatic adhesion of dsRNA to SPc’s surface.

Isothermal titration calorimetry (ITC) is a high-accuracy method for measuring binding affinities, which is a universal method that has broad impact throughout biotechnology [51, 52]. ITC was then performed to further illustrate the interaction between dsRNA and SPc (Fig. 1b). According to the previous interpretation of ITC data [53], a high affinity constant (K_a_) of 5.526 × 10^7^ M^−1^ and a low dissociation constant (K_d_) of 1.810 × 10^–8^ M indicate that there is an effective and strong interaction between SPc and ds*eGFP*, and this interaction is automatic due to the negative ∆G of − 43.9 kJ/mol. The negative ∆H and ∆S suggest that the hydrogen bond and van der Waals force also play an important role in the self-assembly of ds*eGFP*/SPc complex. However, the putative combination site for hydrogen bond is still not known. Based on the chemical structure, multifunctional SPc can be complexed with exogenous substances through various interaction forces such as hydrogen bond and van der Waals force with chitosan, electrostatic interaction with eugenol, thiamethoxam and osthole, and hydrophobic interaction with matrine [54–57]. Different self-assembly mechanisms of SPc with exogenous substances are beneficial for expanding the application area of SPc.

### Efficient protective effects of nanocarriers on dsRNA

**Fig. 2:**
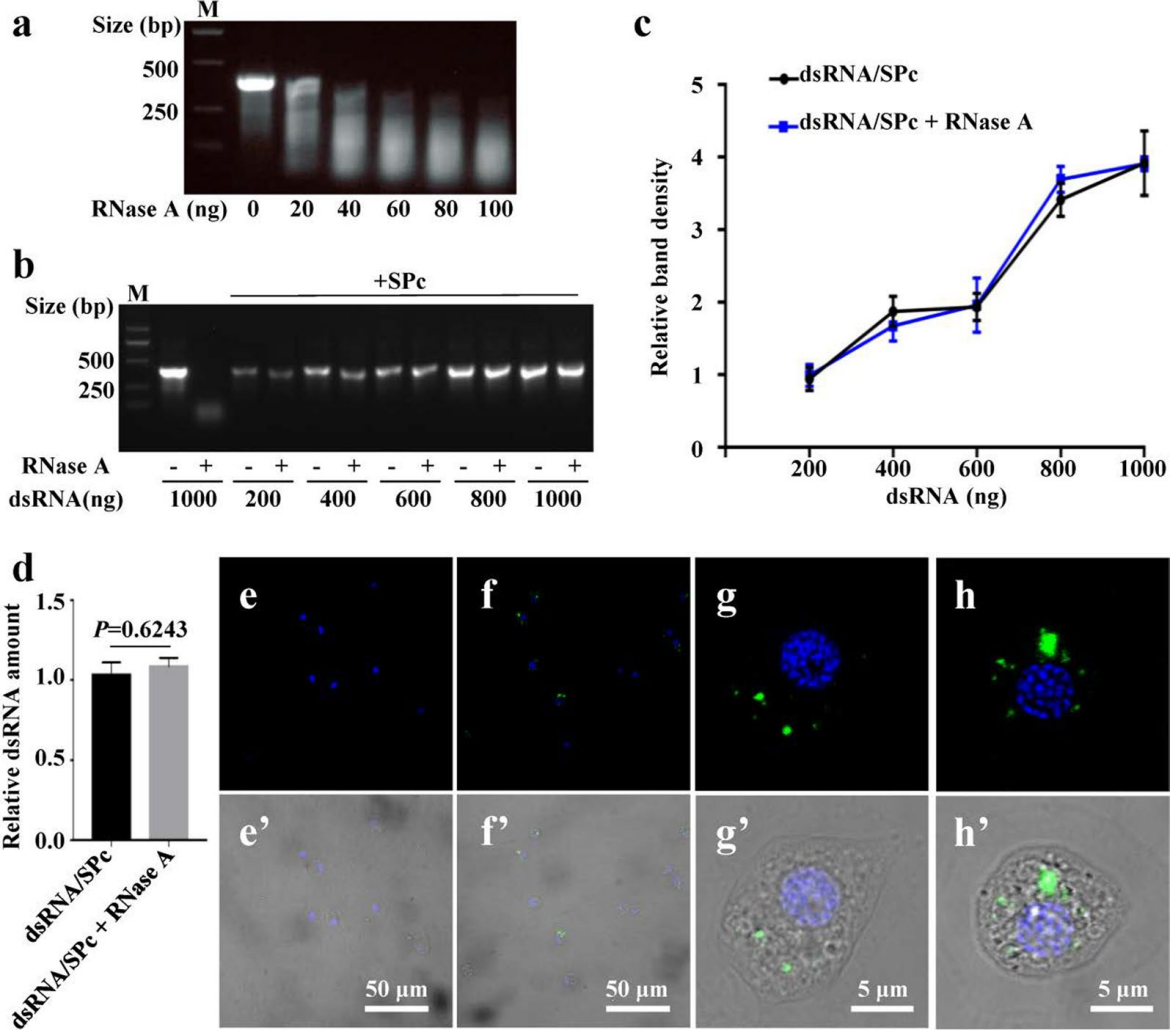
Enhanced stability of SPc-complexed dsRNA. **a** The ds*eGFP* degradation by RNase A. One μg ds*eGFP* was added with RNase A to prepare the reaction solution (ds*eGFP*: 100 ng/μL), and the mixture was incubated for 20 min at 37 °C. M: DNA marker. Gel electrophoresis assay (**b**) and relative band density (**c**) of SPc-complexed ds*eGFP* treated with RNase A. The RNase A was used to treat ds*eGFP*/SPc complex (ds*eGFP*: 1 μg). Then the ds*eGFP*/SPc complex was decomplexed in 0.3% SDS solution. Each treatment was repeated 3 times. **d** Relative dsRNA amount of SPc-complexed ds*eGFP* treated with RNase A. The decomplexed ds*eGFP* was purified and quantified. Each treatment was repeated 3 times. Statistical analysis was conducted using independent *t*-test at the *P* = 0.05 level of significance. **e**–**h’** Fluorescent images of immune cells treated with naked ds*eGFP* (**e**-**e’**) or SPc-complexed ds*eGFP* (**f**–**h’**). The ds*eGFP* and ds*eGFP*/SPc complex were incubated with hemolymph for 3 h (ds*eGFP*: 500 ng). Blue: DAPI. Green: ds*eGFP*. **g**-**g’** Plasmatocyte. **h**–**h’** Granulocyte

The degradation of dsRNA can be very fast [58], which was also confirmed by our results that the ds*eGFP* could be degraded quickly by RNase A (Fig. 2a). Interestingly, there was no significant change in band density of SPc-complexed ds*eGFP* treated with RNase A (Fig. 2b, c). The decomplexed ds*eGFP* was purified and quantified, and the results demonstrated that RNase A could not degrade SPc-loaded dsRNA (Fig. 2d). Similarly, the SPc could also prevent ds*eGFP* from degradation by hemolymph, providing a strong protection (Additional file 1: Fig. S2). Based on the increased particle size of ds*eGFP*/SPc complex compared to SPc (unpublished data), we deduced that the ds*eGFP* might be adhered to SPc’s surface. The improved stability of SPc-loaded ds*eGFP* might be that there were no/less exposed acting sites for degradation by RNAase A. Similar to previous studies, a guanidine-containing polymer is able to protect dsRNA against nucleolytic degradation at pH 11 in gut juice of beet armyworms [28]. Cationic lipids can protect dsRNA from degradation by endonuclease present in Sf9 cells conditioned medium, hemolymph and midgut lumen contents collected from the larvae of *S. frugiperda* [32].

In addition to the fast digestion of free dsRNA by dsRNases, exotic dsRNA is usually unstable in immune cells in hemolymph [59, 60]. Fluorescent ds*eGFP* was synthesized by being labelled by fluorescein-12-UTP through in vitro transcription, which could be detected at 488 nm [61]. Thus, it was used to perform the stability test of SPc-loaded dsRNA in confrontation with immune cells. The combination of SPc with ds*eGFP* did not change the fluorescence intensity of ds*eGFP*, thus the SPc is fit for investigating the fate of nanocarrier-delivered fluorescent dsRNA in immune cells (Additional file 1: Fig. S3). The signal of naked ds*eGFP* was undetectable in immune cells (Fig. 2e-e’), which might be due to the degradation of ds*eGFP* by immune cells/hemolymph and the low penetrability across the cell membrane. Whereas, the fluorescent signal was strongly detectable in immune cells treated with ds*eGFP*/SPc complex (Fig. 2f-f’) that could be still detected in plasmatocyte (Fig. 2g-g’) and granulocyte (Fig. 2h-h’) after incubation for 3 h. The SPc-delivered ds*eGFP* could penetrate the cell membrane less than 30 min, and the current results revealed that the ds*eGFP*/SPc complex could remain stable in immune cells in a period of time.

### SPc-mediated efficient delivery of dsRNA

**Fig. 3:**
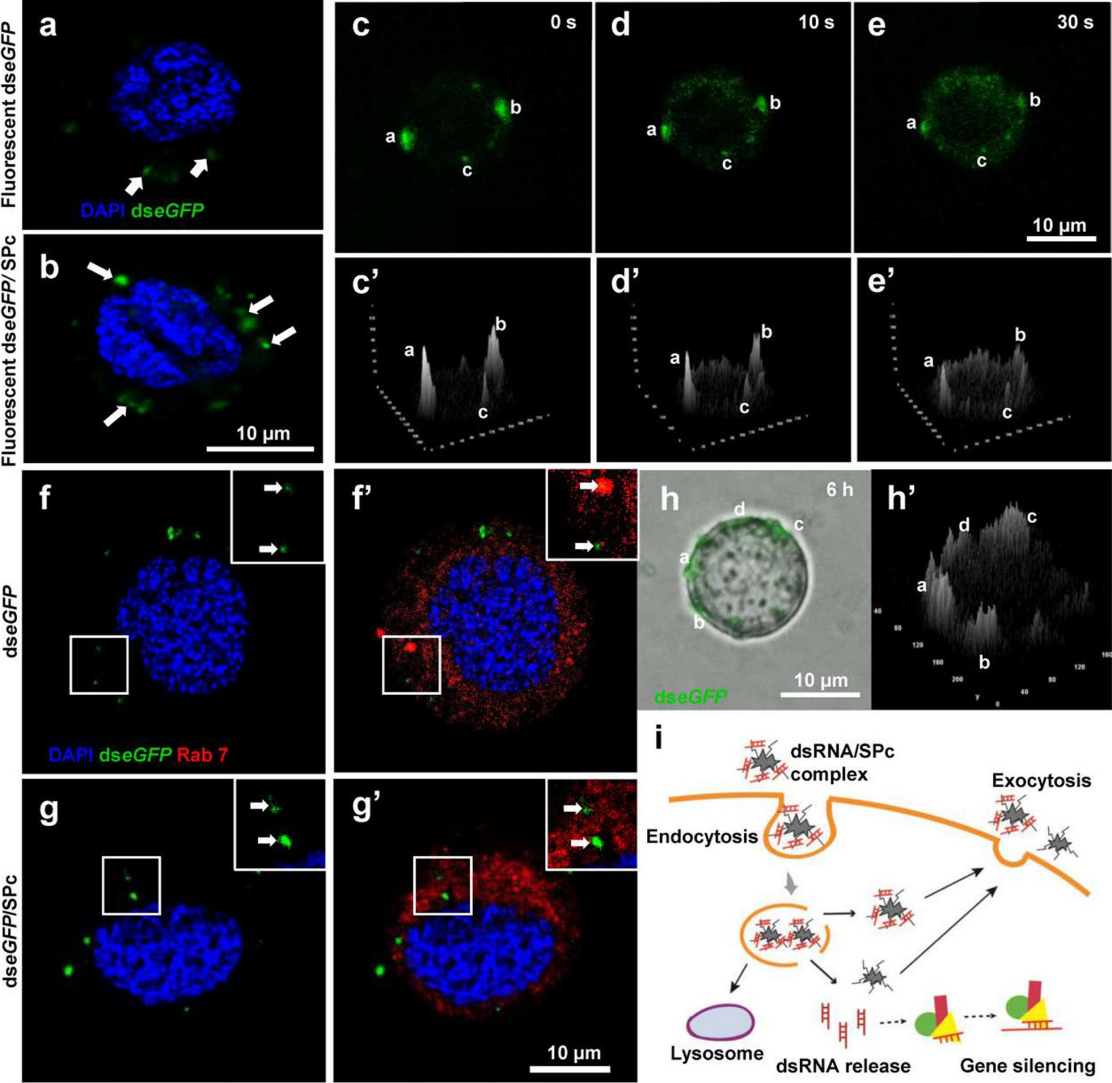
Improved endocytosis and exocytosis of dsRNA/SPc complex. (a-b) Cellular uptake of naked ds*eGFP* (**a**) and ds*eGFP*/SPc complex (**b**). The cells were incubated with ds*eGFP* and ds*eGFP*/SPc complex for 6 h, respectively (ds*eGFP*: 500 ng). Blue: DAPI. Green: ds*eGFP*. (**c**–**e**’) Vesicle release of SPc-delivered ds*eGFP* into cytoplasm. The cells were incubated with ds*eGFP* and ds*eGFP*/SPc complex, respectively (ds*eGFP*: 500 ng) for 6 h. A surface plot test was conducted using ImageJ. Three typical regions were marked. **f**, **g’** Endosomal escape of SPc-delivered dsRNA. The cells incubated with SPc-delivered dsRNA for 6 h were imaged. Red: Rab7 marking the late endosome. **h**–**h’** Potential exocytosis of dsRNA/SPc complex. The cells incubated with SPc-delivered dsRNA for 6 h were re-suspended in fresh medium, and then imaged. **i** Schematic illustration of endocytosis and exocytosis of SPc-delivered dsRNA

Previous study has confirmed that the dsRNA could be taken up by Sf9 cells within 30 min of incubation [61]. In the current study, both ds*eGFP*/SPc complex and naked ds*eGFP* could be taken up by Sf9 cells quickly. However, significant higher fluorescent intensity was observed with ds*eGFP*/SPc complex, and this phenomenon was more obvious after 12 h incubation (fluorescence intensity: 115a.u. vs. 19a.u.), indicating that the SPc could promote the cellular uptake of dsRNA (Fig. 3a, b and Additional file 1: Fig. S4). Compared to dsRNA, delivery of naked siRNA remains a considerable hurdle owing to inefficient cellular uptake [62]. [63] demonstrated that long dsRNA bound to cells and was localized in large puncta in the cell interior, and low-level binding and no obvious internalization of siRNA were observed. As shown in Additional file 1: Fig. S5, siRNA alone exhibited no uptake by Sf9 cells, but the cellular uptake of siRNA was remarkably improved with the help of SPc, suggesting that the SPc could efficiently deliver both long and short dsRNA across the cell membrane. Our previous study has confirmed that the SPc could combine with nucleic acids and the zeta potential of formed complex was still positive [44]. The slight positive electricity of dsRNA/SPc or siRNA/SPc complex was beneficial for its adhesion to cell membrane.

Exogenous substances are commonly taken up through endocytosis, and the endocytic vesicles generated by dsRNA/nanoparticle complex travel along microtubes and subsequently fuse with early endosomes [23, 64]. As shown in Fig. 3c–e’ and Additional file 1: Fig. S6, the fluorescence signal in representative cells was firstly located in the early vesicles close to the cell membrane in dse*GFP*/SPc complex treatment following the cellular uptake, and the complex was then dispersed into the cytoplasm quickly, suggesting that the dsRNA could be released from the vesicles. The dsRNA/SPc complex should exit endosome to avoid the degradation in late endosome (lysosome) [65, 66]. The “proton sponge” hypothesis is the most generally accepted mechanism of endosomal escape, although it is heavily debated [67–69]. Many cationic polymers have a strong buffering capacity over a range of pH between 5 and 7, and the acidic environment in late endosome can lead to the protonation of their amine groups, thereby causing a water influx that leads to endosome lysis [23, 70, 71]. The cells taking up similar amounts of dsRNA and dsRNA/SPc complex were selected to determine the endosomal escape. Compared to naked dsRNA, there was no accumulation of SPc-delivered dsRNA in the late endosomes of representative cells labelled by a specific antibody (Fig. 3f-g’ and Additional file 1: Fig. S7), indicating that the SPc could promote the endosomal escape of dsRNA. Similar to a previous study, lipid formulated dsRNA exhibits reduced accumulation in the endosomes of Sf9 cells [32].

The exocytosis and intercellular spreading of dsRNA are important for systemic RNAi, which seems to be less efficient in lepidopteran species [61, 72]. To directly visualize the potential exocytic vesicles of dse*GFP*/SPc complex, the culture medium containing fluorescent dse*GFP*/SPc complex was immediately replaced with fresh medium after incubation for 6 h. The fluorescent signal was accumulated and located close to the cell membrane (Fig. 3h-h’ and Additional file 1: Fig. S8), which might represent the exocytosis of the complex. Therefore, we deduced that the SPc might promote the excretion and intercellular spreading of dsRNA to improve systemic RNAi although we failed to detect the fluorescence signal outside the cells, which might be due to the rapid diffusion of dsRNA in culture medium. A model was further proposed to illustrate the cellular uptake and intracellular trafficking of SPc-delivered dsRNA (Fig. 3i).

### SPc activates the clathrin-mediated endocytosis for enhanced dsRNA delivery

**Fig. 4:**
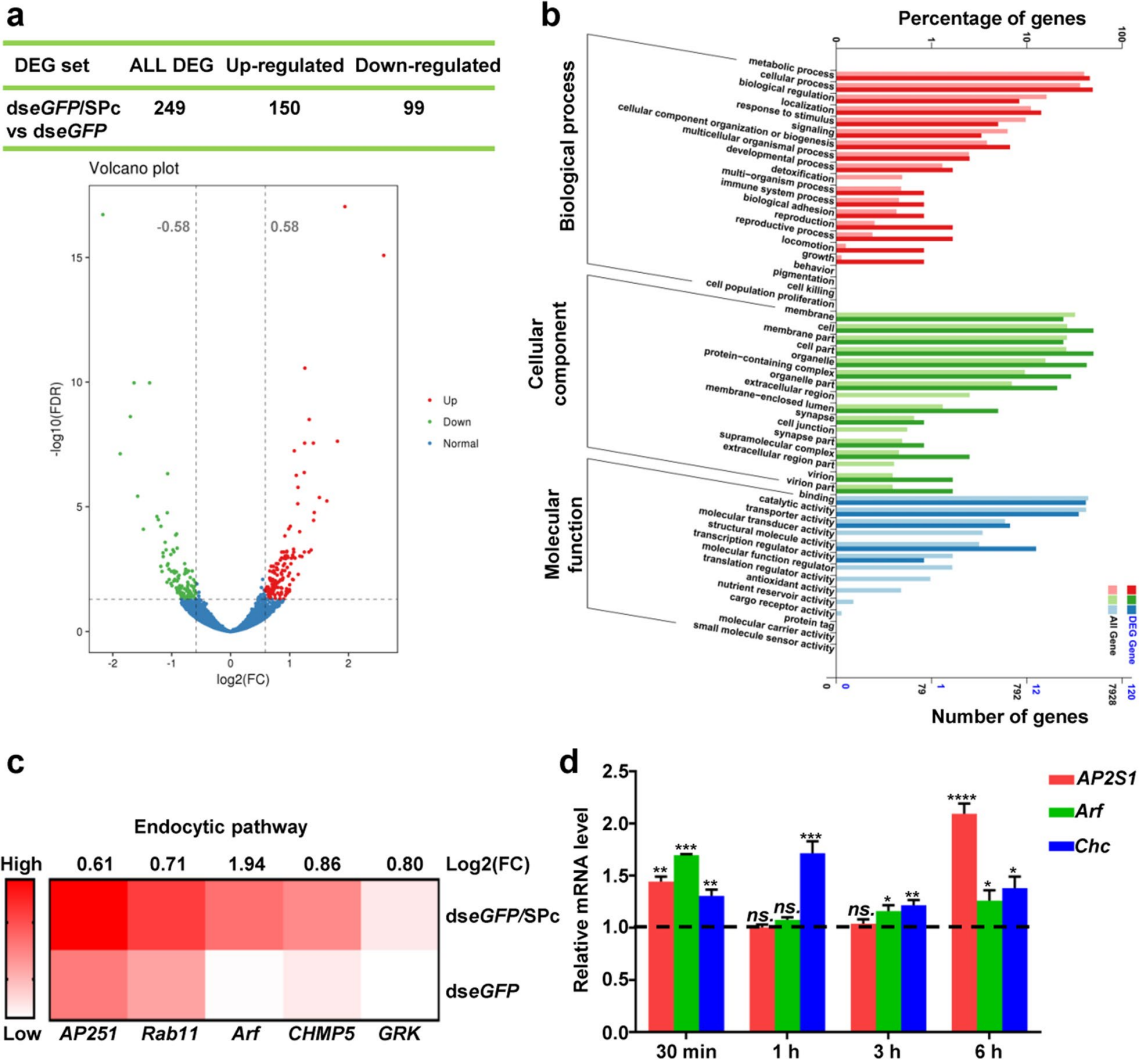
Comparative transcriptome analysis of Sf9 cells treated with naked ds*eGFP* and ds*eGFP*/SPc complex. Analysis of differentially expressed genes (DEGs) with volcano plot (**a**) and GO annotation (**b**). Cells were collected at 6 h after incubation with ds*eGFP* and ds*eGFP*/SPc complex (ds*eGFP*: 500 ng) for RNA extraction. **c** Heatmap of the endocytic pathway. **d** QRT-PCR analysis of the genes related to clathrin-mediated endocytosis. The expression levels of *AP2S1*, *Arf* and *Chc* were determined at various time points after the treatment of ds*eGFP* or ds*eGFP*/SPc complex (ds*eGFP*: 500 ng). Each treatment consisted of 3 replications. The *actin* and *ribosomal protein S15* (*RPS-15*) were used as reference genes. Asterisk indicates significant difference in gene expression between ds*eGFP*/SPc complex and naked ds*eGFP* treatment according to independent *t*-test (ns.: no significant difference, *P < 0.05, **P < 0.01, ***P < 0.001 and ****P < 0.0001)

To identify the crucial regulators during cellular uptake of SPc-delivered dsRNA, transcriptome sequencing was performed in vitro. There were 150 up-regulated genes and 99 down-regulated genes in differentially expressed genes (DEGs). Among them, 77% (116/150) of up-regulated genes and 74% (73/99) of down-regulated genes enriched in the section of log2 (fold change) = [0.58, 1] were further investigated (Fig. 4a). The unigenes were divided into three main categories such as biological process, cellular component and molecular function (Fig. 4b). As expected, differentially expressed genes (DEGs) were enriched in various signaling pathways, and the endocytic pathway was activated by the application of ds*eGFP*/SPc complex significantly. As crucial genes regulating endocytosis and exocytosis, *AP2S1*, *Arf1*, *Rab11*, *CHMP5* and *GRK* were significantly up-regulated in cells treated with dsRNA/SPc complex (Fig. 4c). *AP2S1* encodes the sigma subunit of the Adaptor Protein 2 complex, which drives endocytic vesicle formation at the plasma membrane [73, 74]. ARF protein regulates vesicular traffic and organelle structure by recruiting coat proteins, which plays a crucial role in fundamental biological processes, such as endocytosis, secretion, phagocytosis etc. [75, 76]. Surprisingly, the expression of *Chc* gene, which encodes a major structural polypeptide of the surface lattice of clathrin-coated pits and vesicles [77, 78], was not significant changed. Thus, we further performed a qRT-PCR analysis of *Chc*, *AP2S1* and *Arf1* at various incubation time points (Fig. 4d). Three genes were all up-regulated in cells treated with dsRNA/SPc complex, suggesting that the SPc might promote the cellular uptake of dsRNA by activating clathrin-mediated endocytosis.

**Fig. 5:**
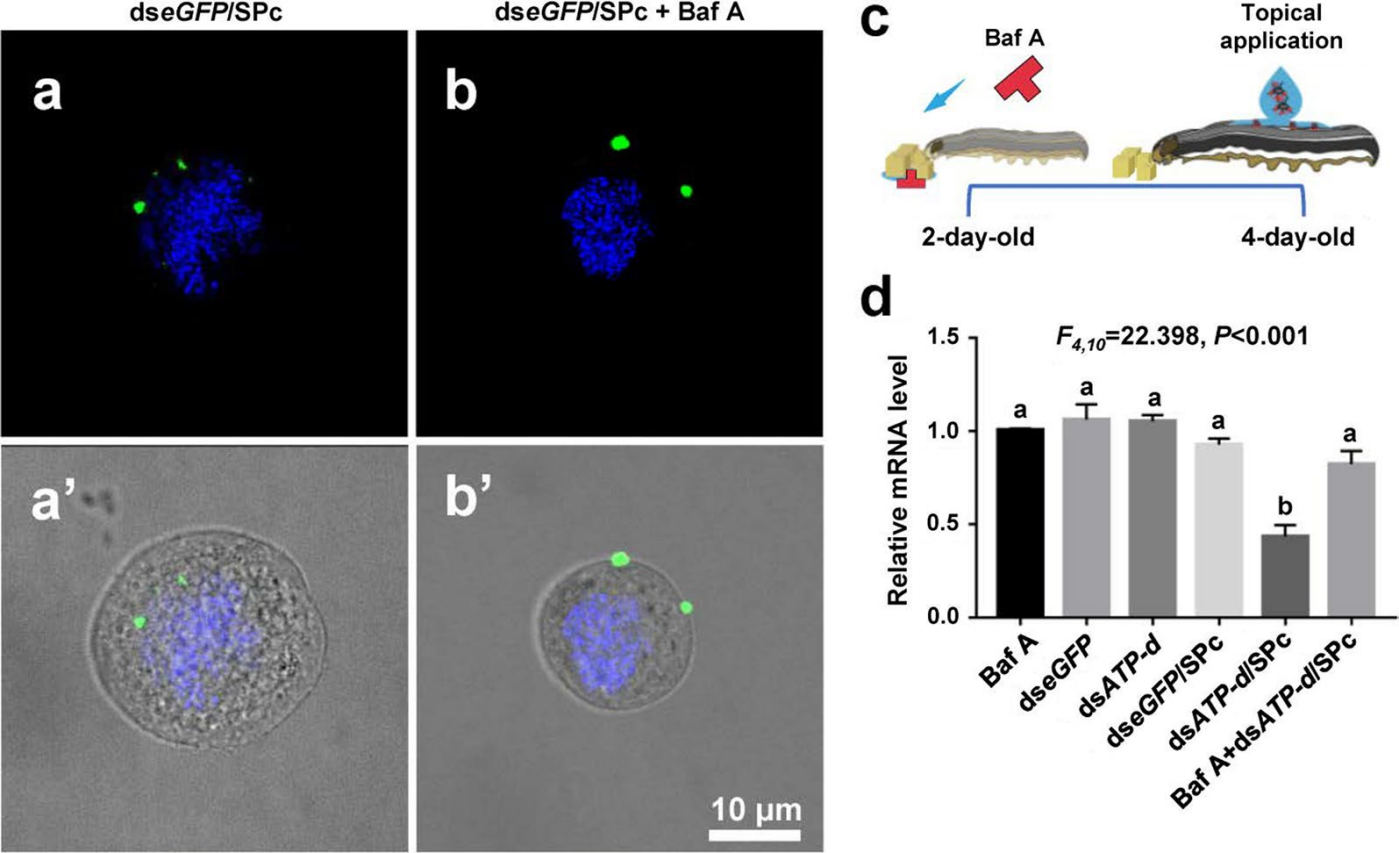
Pharmacological inhibitor blocks the SPc-mediated dsRNA delivery. **a**, **b’** Inhibitor application hindered the cellular uptake of ds*eGFP*/SPc complex. The cells were exposed to 0.2 μM Bafilomycin-A (inhibitor) for 30 min, incubated with ds*eGFP*/SPc complex, and then examined. Blue: DAPI. Green: ds*eGFP*. **c** Schematic diagram of pharmacological assay toward *S. frugiperda*. The 2-day-old larvae were fed with Baf A for 2 days. Then, RNAi was performed using 4-d-old larvae. **d** QRT-PCR analysis of *ATP-d* gene expression in larvae fed with inhibitor. Each treatment consisted of 3 replications. Different letters on columns indicate significant differences (Tukey HSD test, *P* < 0.05)

Previous studies revealed that the clathrin-mediated endocytosis might be involved in RNAi responses of *Drosophila* S2 cells and red flour beetles using pharmacological block [63, 79]. The role of clathrin-mediated endocytosis in the cellular uptake of dsRNA/SPc complex was further confirmed in vitro and in vivo using the similar method. Bafilomycin-A (Baf A) can suppress the transporting protons out of the cell across the plasma membrane, which has been widely applied to inhibit clathrin-mediated endocytosis [63]. From the represent photos, the inhibitor application hindered the cellular uptake of SPc-delivered ds*eGFP* (Fig. 5a, b’). Furthermore, as a housekeeping gene, the *ATP-d* gene identified by our previous study was taken as an example to determine the effects of inhibitor on RNAi efficiency [80]. The SPc-delivered ds*ATP-d* could not down-regulate *ATP-d* gene expression of *S. frugiperda* larvae fed with inhibitor, revealing the ds*ATP-d* lost biological function after inhibitor application (Fig. 5c, d). Our results confirmed that the clathrin-mediated endocytosis was the major pathway for the cellular uptake of SPc-delivered dsRNA.

## Conclusions

In summary, a star polymer (SPc) consisted of a hydrophilic shell with positively-charged tertiary amine in the side chain was synthesized, which was taken as an example to illustrate the mechanism and detailed process of nanoparticle-mediated RNAi. The SPc could assemble with dsRNA spontaneously through electrostatic force, hydrogen bond and van der Waals force. Interestingly, the SPc could protect dsRNA from degradation by RNase A and insect hemolymph remarkably. With the help of SPc, dsRNA could be efficiently taken up by cells, successfully released from early endosome, diffused into the cytoplasm, and transmitted among cells for systemic RNAi. Transcriptome analysis revealed that the SPc could up-regulate some key genes such as *Chc*, *AP2S1* and *Arf1* for activating clathrin-mediated endocytosis pathway. Furthermore, the application of a specific inhibitor hindered the cellular uptake of dsRNA/SPc complex in vitro, and the RNAi effect was also disappeared in vivo**.** Our study thoroughly revealed the process how nanocarrier deliver dsRNA and this mechanism is beneficial for understanding the mechanism of nanocarrier-mediated RNAi, which will support the wide application of nanocarrier-delivered gene for therapy and pest control.

## Methods

### Cell culture and insect rearing

The Sf9 cells from ovaries of fall armyworms (*S. frugiperda*) were cultured in Sf-900 II SFM Medium (Gibco, USA) at 27 °C supplemented with 10% fetal bovine serum, penicillin (100 unit/mL) and streptomycin (100 μg/mL). *S. frugiperda* larvae were fed on an artificial diet bought from Tuidongzhe Biotechnology Co. (China), and reared under a photoperiod of 16 h light: 8 h dark at 25 °C.

### Gel retardation test and isothermal titration calorimetry (ITC) assays

The *enhanced green fluorescent protein* gene (*eGFP*) was selected for dsRNA synthesis using the T7 RiboMAX expression (Promega, USA). All primers (Additional file 2: Table S1) were synthesized by Tsingke (China). The SPc was synthesized according to the methods described by [44]. A gel retardation test was firstly performed to determine the best mass ratio for the combination of dsRNA with nanoparticle. One μg ds*eGFP* was mixed with SPc at various mass ratios, and the mixture (10 μL) was incubated at room temperature for 15 min and then analyzed by agarose gel electrophoresis. To determine the interaction of dsRNA with SPc, the 5.1 μM SPc was titrated with 0.333 μM ds*eGFP* in Nano ITC (TA Instruments Waters, USA). The heats of interaction during each injection were calculated by the integration of each titration peak via the Origin7 software (OriginLab Co., USA). The test temperature was 25 °C, and the ΔG was calculated using the formula of ΔG = ΔH − TΔS.

### Stability test of nanocarrier-complexed dsRNA

To determine the stability of dsRNA, one μg ds*eGFP* was added with RNase A (Sigma-Aldrich, USA) to prepare the reaction solution (ds*eGFP*: 100 ng/μL), and the mixture was incubated for 20 min at 37 °C. The hemolymph collected from 5^th^ instar larvae of *S. frugiperda* [81] was diluted with 1 × PBS and mixed with 1 μg ds*eGFP*, and the mixture was incubated for 1.5 h at room temperature. The agarose gel electrophoresis was used to detect the integrity of dsRNA treated with RNase A or hemolymph.

To investigate the stability of nanocarrier-complexed dsRNA, dsRNA was mixed with SPc at the best mass ratio, respectively. The RNase A was used to treat ds*eGFP*/SPc complex (ds*eGFP*: 1 μg). Then the ds*eGFP*/SPc complex was decomplexed in 0.3% SDS solution and analyzed using agarose gel electrophoresis. The relative band density was determined using ImageJ 1.48v (National Institutes of Health, USA). Meanwhile, decomplexed ds*eGFP* was purified using MEGAclear Kit (Thermo Fisher Scientific, USA), and quantified using NanoDrop 2000 spectrophotometer (Thermo Fisher Scientific). Naked ds*eGFP* was applied as control, and each treatment was repeated 3 times. The stability of ds*eGFP*/SPc complex treated with hemolymph was analyzed similarly.

For the stability test of nanocarrier-complexed dsRNA in confrontation with immune cells, 100 μL of hemolymph was added with 400 μL Sf-900 II SFM Medium. Fluorescent ds*eGFP* was synthesized using Fluorescein RNA labeling Mix (Roche Diagnostics, USA) according to the manufacturer’s protocol. The dsRNA was labelled by fluorescein-12-UTP through in vitro transcription, which could be detected at 488 nm [61]. The fluorescent ds*eGFP* was mixed with SPc at the best mass ratio. Changes in fluorescence intensity of ds*eGFP* complexed with SPc were then tested using NanoDrop 2000 spectrophotometer at 488 nm. The ds*eGFP* and ds*eGFP*/SPc complex were incubated with hemolymph for 3 h, respectively (ds*eGFP*: 500 ng). The cells were washed, fixed with 4% paraformaldehyde, deposited on the slides using an Antifade Mounting Medium with DAPI (Vector Laboratories, USA), and then examined using a confocal microscope (Leica SP8, Germany).

### Cellular uptake and intracellular trafficking of SPc-delivered dsRNA/siRNA

Fluorescent ds*eGFP* and si*eGFP* (GenePharma Co., China) were used to determine the cellular uptake of SPc-delivered dsRNA/siRNA. The cells were treated with ds*eGFP*, ds*eGFP*/SPc complex, si*eGFP* and si*eGFP*/SPc complex, respectively (ds*eGFP*: 500 ng; si*eGFP*: 280 ng). Cells were imaged using a confocal microscope at various time points after incubation. The time-lapse imaging for tracing ds*eGFP*/SPc complex was conducted using an inverted fluorescence microscope (Olympus, Japan) after 6 h of incubation, followed by a qualification of the intracellular fluorescence intensity by Surface Plot Program using ImageJ 1.48v at various time points. The fluorescent intensity was determined using ten cells as ten replications for ds*eGFP* delivery.

To investigate the intracellular trafficking of SPc-delivered dsRNA, cells incubated for 6 h were re-suspended in fresh medium for 30 min, and then imaged using an inverted fluorescence microscope. The intracellular fluorescence intensity was determined using Surface Plot Program similarly. Meanwhile, cells incubated with dsRNA/SPc complex and dsRNA for 6 h were deposited on the slides, fixed with 4% paraformaldehyde, treated with 0.1% Triton X-100, and then blocked with 0.2% bovine serum albumin. Blocked samples were incubated with primary antibody mouse anti-Rab 7 (1:200, DSHB, USA) overnight, incubated with secondary antibody goat anti-mouse Cy5 (1:200, Jackson, USA) for 1.5 h, and then examined using a confocal microscope.

### Transcriptome analysis

Cells were collected at 6 h after incubation with ds*eGFP* and ds*eGFP*/SPc complex (ds*eGFP*: 500 ng), respectively. Total RNA was extracted from three biological replicates using the TRNzol formulation (TIANGEN, China). The transcript libraries were constructed via Illumina HiSeq sequencing platform. Raw reads containing connectors and with low-quality (Q ≤ 10) were removed. The resulting clean reads were assembled using Trinity software [82]. TopHat2 was used to achieve the sequence alignment of clean reads with the reference genome (http://www.insect-genome.com/Sfru/) [83]. BLASTX was used to compare unigene sequences with the Non-Redundant protein sequence database (NR) and Swiss-Prot database for annotation of unigenes. The expression level of each transcript was presented by FPKM value. Deseq was applied for differential expression analysis between transcripts, and fold change ≥ 1.5 and FDR < 0.05 were screen conditions [84].

The expression levels of endocytosis-related gene *AP2S1*, *Arf* and *Chc* were determined using quantitative real-time PCR (qRT-PCR) at various time points after the treatment of ds*eGFP* or ds*eGFP*/SPc complex (ds*eGFP*: 500 ng). All primers for qRT-PCR were shown in Additional file 2: Table S2. The qRT-PCR was performed with Step One Plus Real-Time PCR system (Applied Biosystems, USA) using Power SYBR® Green Master Mix (Applied Biosystems). The *actin* and *ribosomal protein S15* (*RPS-15*) were selected as reference genes, and the relative mRNA levels of target genes were normalized to the abundance of two genes using the 2^−∆∆CT^ methods [85].

### Pharmacological inhibitors of SPc-delivered dsRNA

Bafilomycin-A (Baf A) (MCE, USA) has been widely used to inhibit clathrin-mediated endocytosis to determine the major pathway for SPc-mediated cellular uptake [63]. For in vitro test, the cells were exposed to 0.2 μM Baf A (MCE, USA) for 30 min, treated with fluorescent ds*eGFP*/SPc complex, deposited on the slides using an Antifade Mounting Medium with DAPI, and then examined using a confocal microscope. For in vivo test, the *V-type proton ATPase subunit d* (*ATP-d*) identified by our previous study was taken as an example to determine the effects of inhibitor on RNAi efficiency [80]. The ds*ATP-d* was mixed with SPc and 1% volume of surfactant Alkyl Polyglucoside (Wanhua, China) to prepare the ds*ATP-d*/SPc complex formulation (dsRNA and SPc: both 1 μg/μL). The 2-day-old larvae of *S. frugiperda* were fed with Baf A for 2 d, and the 0.5 μL formulation was applied directly on the notum of 4-day-old larvae. The relative mRNA level of *ATP-d* was evaluated using qRT-PCR at 24 h after the topical application. Each treatment consisted of 3 replications.

### Statistical analysis

The ANOVA with Tukey HSD test was conducted using SPSS 20.0 (SPSS Inc., USA) at the *P* = 0.05 level of significance. A two-tailed Student’s *t*-test was performed using Prism 7.0 (GraphPad Software Inc., USA) at the *P* = 0.05 level of significance. The descriptive statistics were shown as the mean value and standard errors of the mean.

## Supplementary Information

**Additional file 1: Figure S1.** Synthetic procedure of SPc. **Figure S2.** Enhanced stability of SPc-complexed dsRNA treated with insect hemolymph. **Figure S3.** Changes in fluorescence intensity of fluorescent dsRNA complexed by SPc. **Figure S4.** Cellular uptake of naked dsRNA and dsRNA/SPc complex. **Figure S5.** Cellular uptake of naked siRNA and siRNA/SPc complex. **Figure S6.** Cytoplasm release of ds*eGFP*/SPc vesicle in one cell by real-time imaging. **Figure S7.** Endosomal escape of SPc-delivered dsRNA. **Figure S8.** SPc-mediated exocytosis of dsRNA.

**Additional file 2: Table S1.** Primers for dsRNA synthesis. **Table S2.** Primers for quantitative real-time PCR (qRT-PCR).

## Data Availability

The datasets used and/or analyzed during the current study are available from the corresponding author on reasonable request.
