# Supplementary material for: Visualization of the process of a nanocarrier-mediated gene delivery: stabilization, endocytosis and endosomal escape of genes for intracellular spreading
Source: J Nanobiotechnology. 2022 Mar 9;20:124. doi: 10.1186/s12951-022-01336-6 (PMC8905852; doi:10.1186/s12951-022-01336-6)
Supplement: Supplementary file 1 — Additional file 1: Figure S1. Synthetic procedure of SPc. Figure S2. Enhanced stability of SPc-complexed dsRNA treated with insect hemolymph. Figure S3. Changes in fluorescence intensity of fluorescent dsRNA complexed by SPc. Figure S4. Cellular uptake of naked dsRNA and dsRNA/SPc complex. Figure S5. Cellular uptake of naked siRNA and siRNA/SPc complex. Figure S6. Cytoplasm release of dseGFP/SPc vesicle in one cell by real-time imaging. Figure S7. Endosomal escape of SPc-delivered dsRNA. Figure S8. SPc-mediated exocytosis of dsRNA. [file 12951_2022_1336_MOESM1_ESM.docx]

**Visualization of the process of a nanocarrier-mediated gene delivery: Stabilization, endocytosis and endosomal escape of genes for intracellular spreading**

Zhongzheng Ma^1,2†^, Yang Zheng^3†^, Zijian Chao^1^, Hongtao Chen^4^, Yunhui Zhang^1^, Meizhen Yin^4^, Jie Shen^1^ and Shuo Yan^1*^

^*^Correspondence: yanshuo2011@foxmail.com

^†^Zhongzheng Ma and Yang Zheng have contributed equally to this work

^1^ Department of Plant Biosecurity and MOA Key Laboratory of Pest Monitoring and Green Management, College of Plant Protection, China Agricultural University, Beijing 100193, P. R. China

^2^ Institute of Plant and Environmental Protection, Beijing Academy of Agricultural and Forestry Sciences, Beijing 100097, P. R. China

^3^ College of Horticulture and Plant Protection, Yangzhou University, Yangzhou 225002, Jiangsu, P. R. China

^4^ State Key Laboratory of Chemical Resource Engineering, Beijing Lab of Biomedical Materials, Beijing University of Chemical Technology, Beijing 100029, P. R. China

**
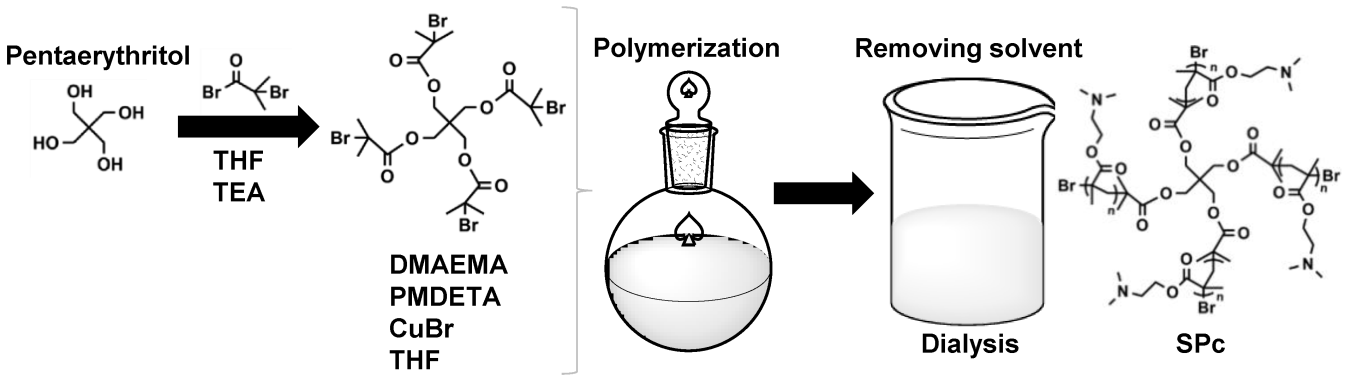
**

**Figure S1.** Synthesis route of SPc. The SPc was synthesized using commercially available pentaerythritol to construct the star initiator Pt-Br, which was further polymerized with DMAEMA. The solvent THF was removed, and dialysis was then conducted to purify the crude product. The SPc was finally obtained as white powder after freeze-drying.

**
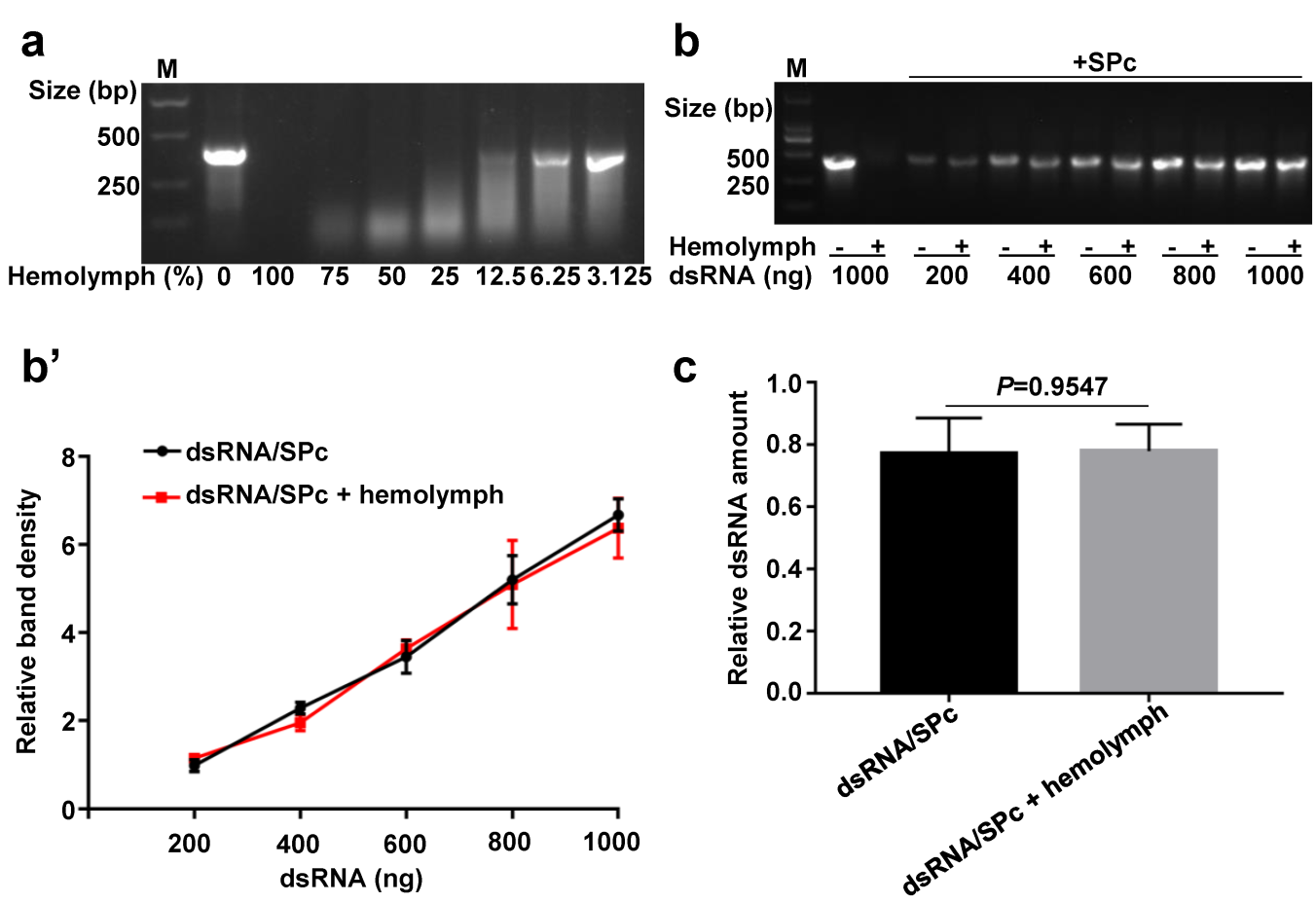
**

**Figure S2.** Enhanced stability of SPc-complexed dsRNA treated with insect hemolymph. (a) The ds*eGFP* degradation by the hemolymph of *S. frugiperda*. One μg ds*eGFP* was incubated with various dilutions of hemolymph for 1.5 h at room temperature. M: DNA Marker. (b-b’) Gel electrophoresis assay (b) and relative band density (b’) of SPc-complexed ds*eGFP* treated with the hemolymph of *S. frugiperda*. Different amounts of ds*eGFP*/SPc complex were incubated with 12.5% of hemolymph for 1.5 h at room temperature. The ds*eGFP*/SPc complex was decomplexed in 0.3% SDS solution and analyzed. The relative band density was determined using ImageJ. Each treatment was repeated 3 times. (C) Relative dsRNA amount of SPc-complexed ds*eGFP* treated with the hemolymph of *S. frugiperda*. The decomplexed ds*eGFP* was purified and quantified. Each treatment was repeated 3 times. Statistical analysis was conducted using independent *t*-test at the *P*=0.05 level of significance.

**
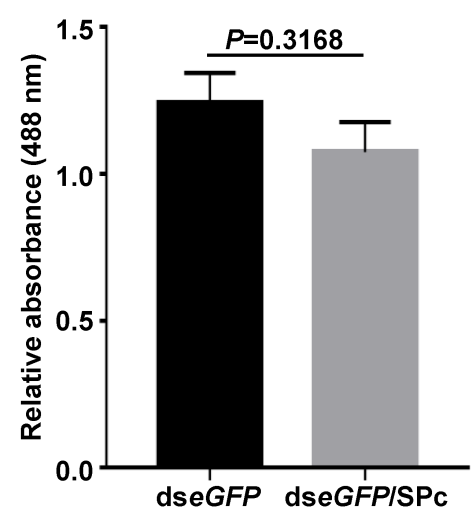
**

**Figure S3.** Changes in fluorescence intensity of dsRNA complexed by SPc. The ds*eGFP* was mixed with SPc at the mass ratio of 1:1 (final concentration for ds*eGFP*: 100 ng/μL). The absorbance comparison of ds*eGFP* and ds*eGFP*/SPc complex at 488 nm was conducted using independent *t*-test at the *P*=0.05 level of significance.

**
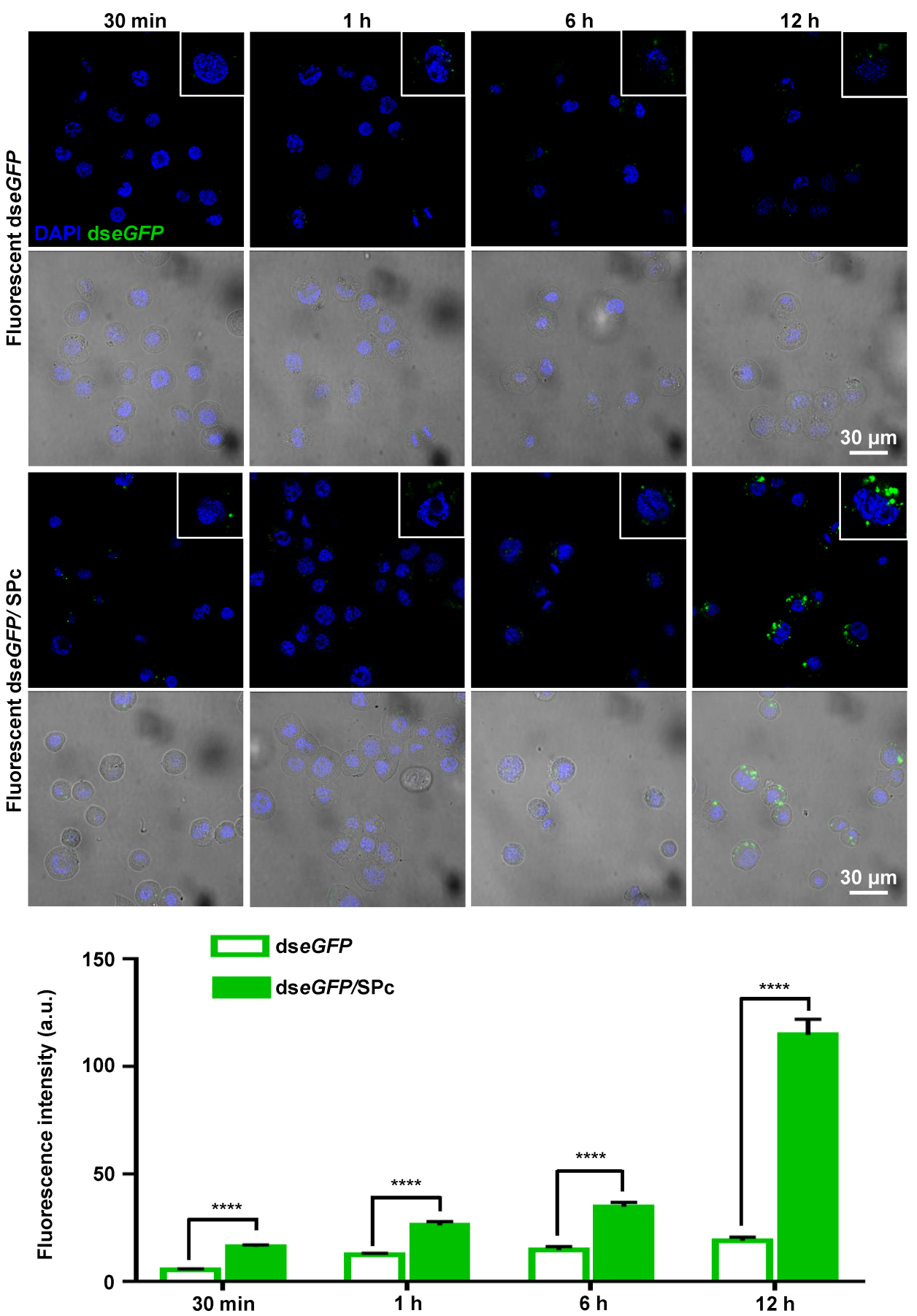
**

**Figure S4.** Cellular uptake of naked dsRNA and dsRNA/SPc complex. The fluorescent ds*eGFP* was mixed with SPc at the mass ratio of 1:1. The cell medium was added with fluorescent ds*eGFP* and ds*eGFP*/SPc complex, respectively (ds*eGFP*: 500 ng). Blue: DAPI. Green: ds*eGFP*.

**
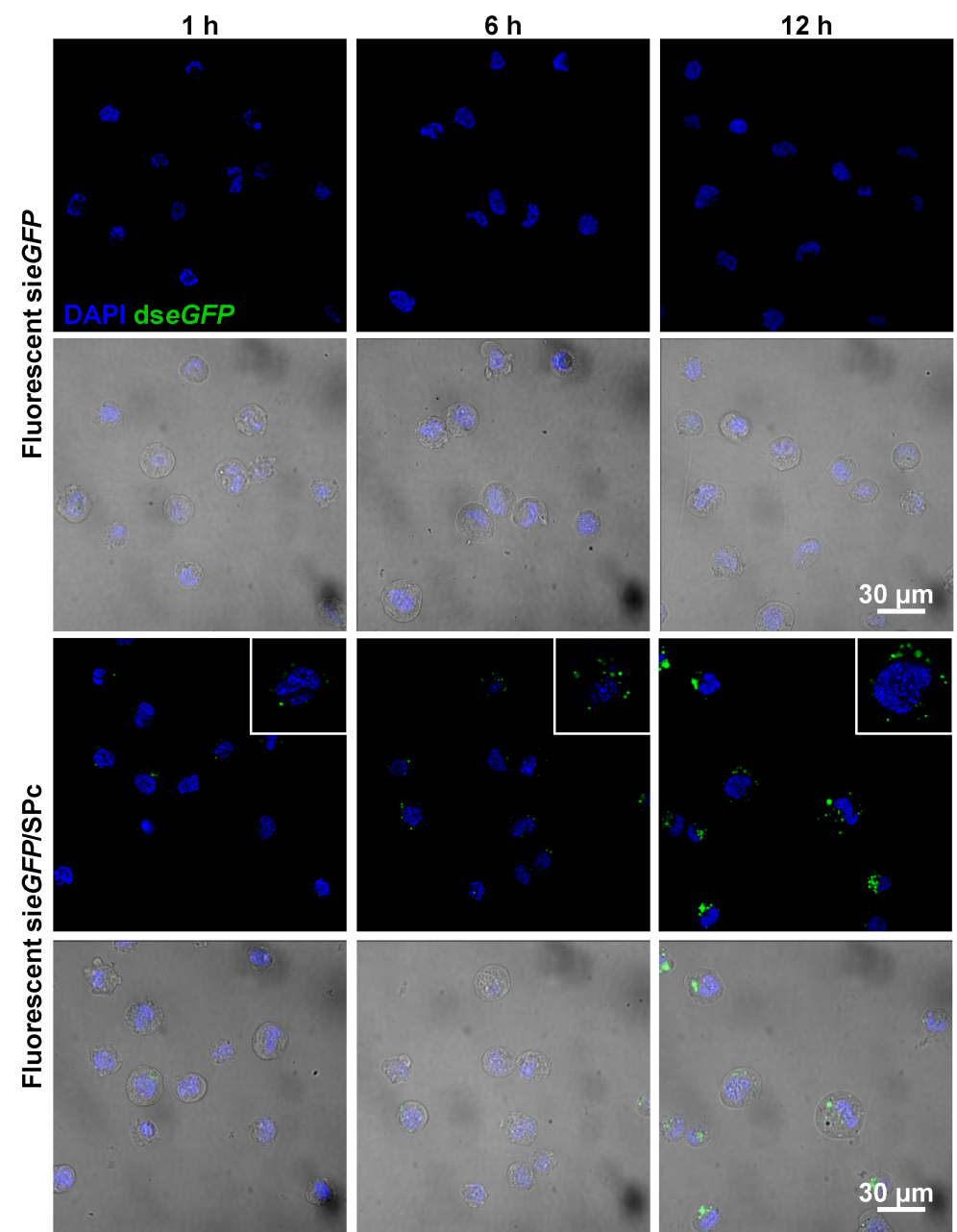
**

**Figure S5.** Cellular uptake of naked siRNA and siRNA/SPc complex. The fluorescent si*eGFP* was mixed with SPc at the mass ratio of 1:1. The cell medium was added with fluorescent si*eGFP* and si*eGFP*/SPc complex, respectively (si*eGFP*: 500 ng). Blue: DAPI. Green: si*eGFP*.


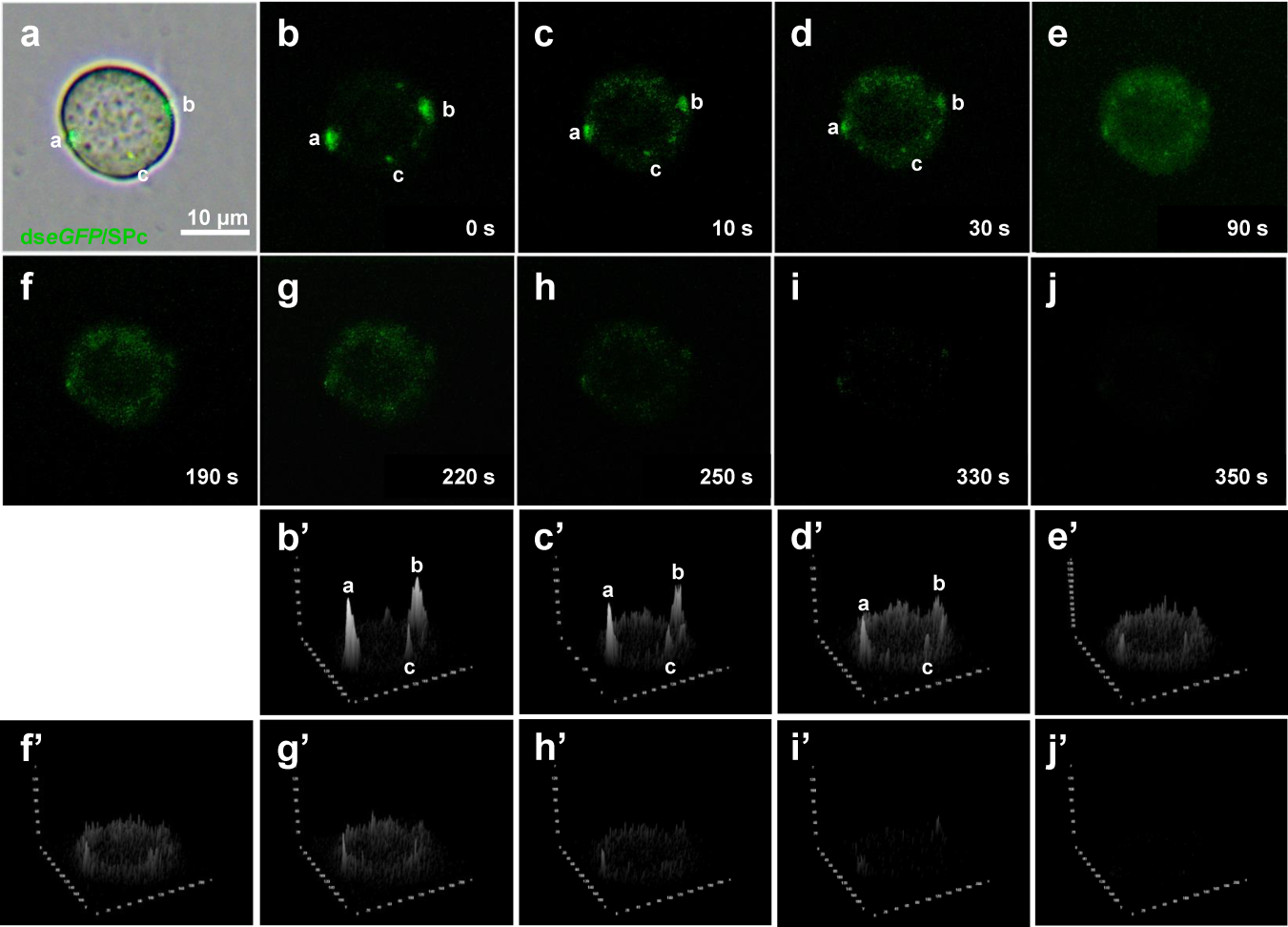


**Figure S6.** Cytoplasm release of ds*eGFP*/SPc vesicle in one cell by real-time imaging. The fluorescent ds*eGFP* was mixed with SPc at the mass ratio of 1:1. The cells were incubated with ds*eGFP* and ds*eGFP*/SPc complex, respectively (ds*eGFP*: 500 ng) for 6 h. A surface plot test was conducted using ImageJ. Three typical regions were marked.


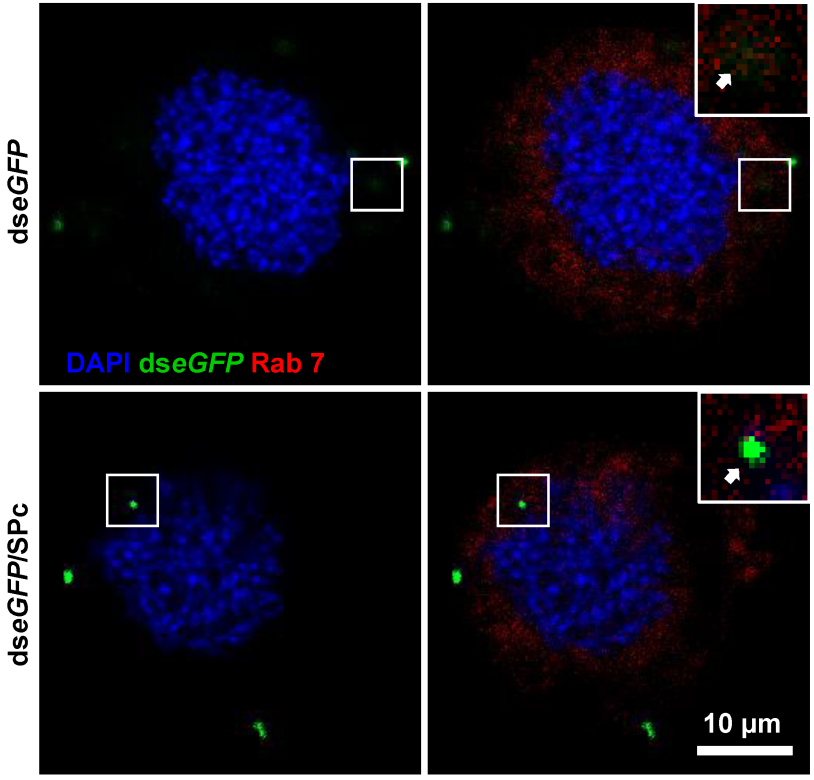


**Figure S7.** Endosomal escape of SPc-delivered dsRNA. The cells incubated with SPc-delivered dsRNA for 6 h were imaged. Red: Rab7 marking the late endosome.

**
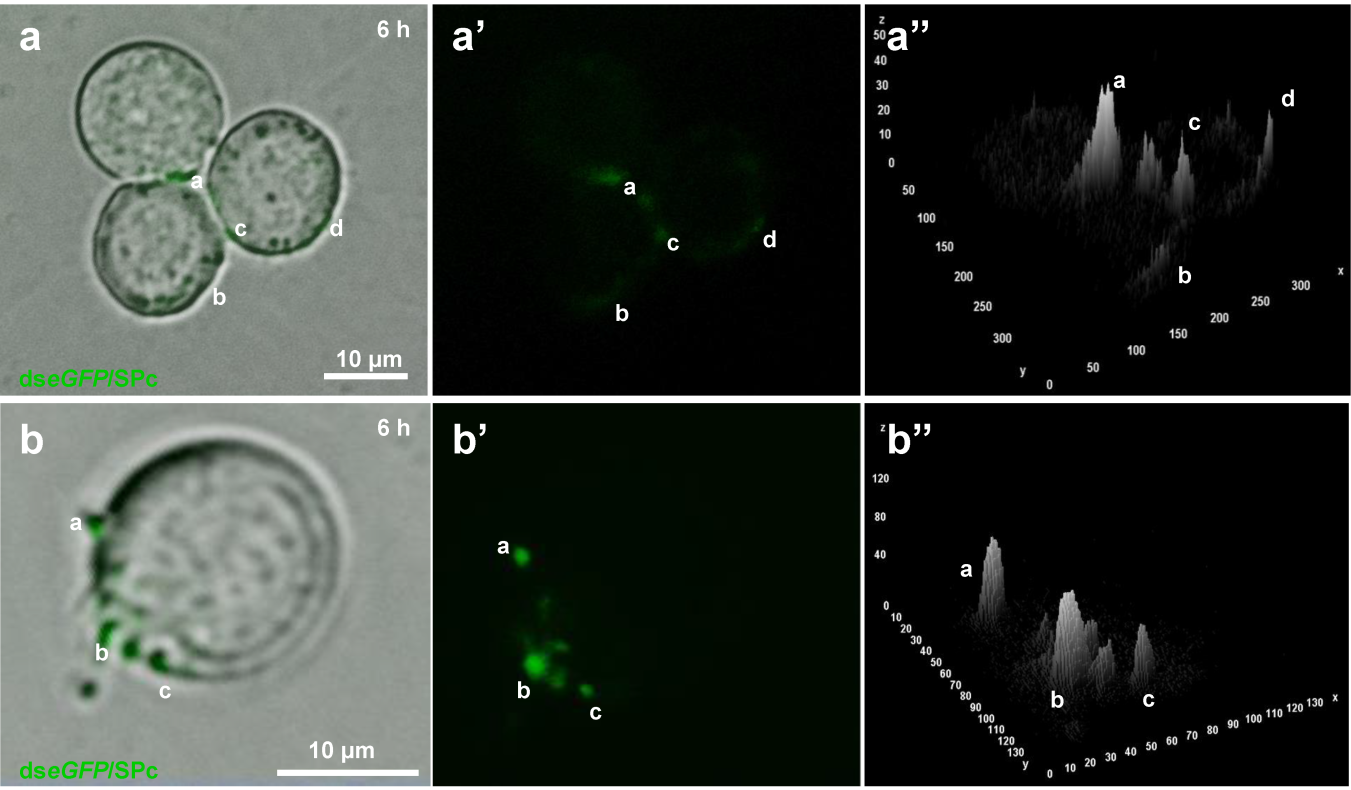
**

**Figure S8.** Potential exocytosis of dsRNA/SPc complex. The cells incubated with dsRNA/SPc complex for 6 h were re-suspended in fresh medium, and then imaged. The fluorescent signal of ds*eGFP*/SPc complex was accumulated and located close to the cell membrane. Two groups of representative photographs were provided. A surface plot test was conducted using ImageJ. Typical regions were marked.
