# Supplementary material for: Visualization of the process of a nanocarrier-mediated gene delivery: stabilization, endocytosis and endosomal escape of genes for intracellular spreading
Source: J Nanobiotechnology. 2022 Mar 9;20:124. doi: 10.1186/s12951-022-01336-6 (PMC8905852; doi:10.1186/s12951-022-01336-6)
Supplement: Supplementary file 2 — Additional file 2: Table S1. Primers for dsRNA synthesis. Table S2. Primers for quantitative real-time PCR (qRT-PCR). [file 12951_2022_1336_MOESM2_ESM.docx]

**Visualization of the process of a nanocarrier-mediated gene delivery: Stabilization, endocytosis and endosomal escape of genes for intracellular spreading**

Zhongzheng Ma^1,2†^, Yang Zheng^3†^, Zijian Chao^1^, Hongtao Chen^4^, Yunhui Zhang^1^, Meizhen Yin^4^, Jie Shen^1^ and Shuo Yan^1*^

^*^Correspondence: yanshuo2011@foxmail.com

^†^Zhongzheng Ma and Yang Zheng have contributed equally to this work

^1^ Department of Plant Biosecurity and MOA Key Laboratory of Pest Monitoring and Green Management, College of Plant Protection, China Agricultural University, Beijing 100193, P. R. China

^2^ Institute of Plant and Environmental Protection, Beijing Academy of Agricultural and Forestry Sciences, Beijing 100097, P. R. China

^3^ College of Horticulture and Plant Protection, Yangzhou University, Yangzhou 225002, Jiangsu, P. R. China

^4^ State Key Laboratory of Chemical Resource Engineering, Beijing Lab of Biomedical Materials, Beijing University of Chemical Technology, Beijing 100029, P. R. China

**Table S1.** Primers for dsRNA synthesis.

| Primer | Sequence |
| --- | --- |
| T7ATP-d-F | TAATACGACTCACTATAGGACTCTACACCAGAGGCCCAT |
| T7ATP-d-R | TAATACGACTCACTATAGGCAGCTTAGCACGGTCATCCT |
| T7eGFP-F | TAATACGACTCACTATAGGCACAAGTTCAGCGTGTCCG |
| T7eGFP-R | TAATACGACTCACTATAGGGTTCACCTTGATGCCGTTC |

The sequences of T7 promoter are underlined with red.

**Table S2.** Primers for quantitative real-time PCR (qRT-PCR).

| Primer | Sequence | PCR efficiency (%) |
| --- | --- | --- |
| qArf1-F | AACTGGTACATCCAGGCGAC | 92.45 |
| qArf1-R | GGCGTTCTTCAGTTGGTTGG |  |
| qAP2S1-F | TGTGAACTGGACCTGGTGTT | 91.04 |
| qAP2S1-R | AGGAGTTGAGCATGAGGAGC |  |
| qChc-F | AAGGTGGCGAACATGGAACT | 90.14 |
| qChc-R | AGAATGTGACGGCTCTGGTG |  |
| actin-F | TAACGAGAGGTTCCGTTGCC | 90.92 |
| actin-R | GTGTTGGCGTACAGGTCCTT |  |
| RPS15-F | ACAATCATCCCGCCGTCATT | 92.43 |
| RPS15-R | GACCTCAGCCATGTTTCCGA |  |
| qATP-d-F | AGGATGACCGTGCTAAGCTG | 98.60 |
| qATP-d-R | CAGCAACAGCCTTAACCTGC |  |
